# Diabetes and bacterial co-infection are two independent risk factors for respiratory syncytial virus disease severity

**DOI:** 10.3389/fmed.2023.1231641

**Published:** 2023-11-01

**Authors:** Hakan Sivgin, Sirin Cetin, Ayse Ulgen, Wentian Li

**Affiliations:** ^1^Department of Internal Medicine, Faculty of Medicine, Tokat GaziosmanPasa University, Tokat, Türkiye; ^2^Department of Biostatistics, Faculty of Medicine, Amasya University, Amasya, Türkiye; ^3^Department of Biostatistics, Faculty of Medicine, Girne American University, Karmi, Cyprus; ^4^Department of Mathematics, School of Science and Technology, Nottingham Trent University, Nottingham, United Kingdom; ^5^Department of Applied Mathematics and Statistics, Stony Brook University, Stony Brook, NY, United States; ^6^The Robert S. Boas Center for Genomics and Human Genetics, The Feinstein Institutes for Medical Research, Northwell Health, Manhasset, NY, United States

**Keywords:** respiratory syncytial virus (RSV), diabetes mellitus, chronic medical condition, bacterial coinfection, disease severity

## Abstract

Diabetes mellitus (DM) is common among older adults hospitalized with lower respiratory tract infection, yet information on the impact of DM on disease severity is limited. This study retrospectively analyzed 46 Turkish patients infected with respiratory syncytial virus (RSV), with information on their comorbidities, co-infection status, and symptoms. Patients are grouped into four severity levels from mild to severe, according to lung parenchymal infiltration status and oxygen level. Similar to previously published studies, we found that comorbidities of diabetes, heart failure, hypertension, co-infection of any type, bacterial co-infection, and age are associated with the disease severity. Cough is the most common symptom (89%) followed by fever (26%) and myalgia, dyspnea, and weakness (around 20%). Using a second-order analysis (two-variable regression), we identified two independent risks for disease severity, the first is represented by diabetes, and the second is represented by bacterial co-infection. We observed two patients whose more severe symptoms were not associated with an older age, but associated with a combination of diabetes and bacterial co-infection. To confirm the true causality from the statistical correlation, further studies are needed.

## Introduction

Lower respiratory tract infections (LRTIs) cause substantial morbidity and mortality globally for both adults (1) and children (2–6). In 2019, there were more than 500 million incidents of LRTI globally (7) and in 2016, there were more than 2 million LRTI-associated deaths (8). These deaths include more than 650,000 children younger than age five and more than one million seniors over the age of 70 (8). The typical medical diagnoses that belong to LRTI include bronchitis, bronchiolitis, and pneumonia. Among the recent coronavirus pandemics, Middle Eastern Respiratory Syndrome (MERS) and Severe Acute Respiratory Syndrome (SARS) (9) have a higher percentage of patients with LRTI, whereas the coronavirus disease of 2019 (COVID-19) (10) infects the lower respiratory system only in a smaller proportion of patients, who tend to have more severe symptoms.

Etiological factors for LRTIs can be bacterial, viral, or fungal. Among viral infections, besides coronavirus, influenza (A or B) and respiratory syncytial virus (RSV) are among the most common causes of viral LRTIs during typical (i.e., non-pandemic) endemic seasons. Influenza causes an estimated one billion infections and RSV is estimated to cause hundreds or tens of millions of acute respiratory infections globally each year. During the COVID-19 pandemic period, however, there was a sharp drop in influenza (11) and bronchiolitis (12), which could be a consequence of quarantine measures (13).

About 60% to 80% of acute respiratory infections in children/infants are caused by RSV, but only 10% or so in adults. Even with this lower percentage, the clinical burden of LRTI in adults caused by RSV is underestimated (14–18). RSV-caused LRTI may result in admission to an intensive care unit (ICU) and death, comparable to those caused by influenza (19–21).

Age is the first factor to consider in assessing the risk of LRTI caused by RSV: both younger children (22, 23) and older seniors (24–27) are vulnerable groups. Besides age, other medical conditions can cause RSV-infected patients to have LRTI and, thus more severe symptoms. For example, cancer patients (28), immunocompromised individuals (29), and those with underlying respiratory and/or cardiovascular comorbidities belong to high-risk groups. Other risk factors (for RSV patients having LRTI) include chronic pulmonary diseases and disability, etc. (30).

We are more interested in the effect of common chronic medical conditions on RSV patients. Take the example of diabetes, not only is it much more common than those rare medical conditions, but it is also common in middle-aged populations. There have been attempts to use diabetic medication to treat RSV patients (31). There is also research interest in how other respiratory tract infections from other viruses and bacteria, such as M. tuberculosis, influenza, and SAR-Cov-2 are impacted by the diabetic condition (32–37). To date, there is limited information on infection-diabetes or lung-diabetes interaction (38, 39) and the mechanisms of potential interplay are poorly understood. The present study was also more focused on age groups not in the extremes, i.e., middle-aged adults (e.g., age 50–64) (40).

In this work, we analyzed medical information from a small group of RSV patients in Tokat, Turkey, to show that diabetes is a very significant risk factor for disease severity. We also investigated the joint impact of diabetes and superbacterial infection on the severity of LRTI. Although more samples are needed to independently validate our findings, our power analysis indicated that our sample size was large enough to detect the observed signal, and it could point to directions where precautionary actions might be taken to save lives.

The standard Methods/Data, Results, and Discussion sections are organized in the following subsections: we first provide summary statistics from our data, including disease severity, comorbidities, symptoms, co-infection, the length of time symptoms lasted, etc. We then provide second-order statistics, i.e., correlation among factors by linear and logistic regression, and identify factors that are associated with the disease severity, extending the single-variable regression. We then undertook two-variable linear regression to identify two factors that jointly contribute to the disease severity; to untangle the confounding relationship between comorbidities and age, we examine the age of onset of comorbidities in more detail, before examining the age of onset of co-infection, as well as the age of onset of bacterial coinfection plus another co-morbidity more carefully.

## Data and methods

### RSV patient data collection

The study was conducted during three consecutive seasons between 2020 and 2023 at Gaziosmanpasa Universty Hospital in Tokat, Tüurkiye. All participants or guardians provided informed consent. The University of Tokat Gaziosmanpasa Faculty Of Medicine Research Subjects Review Board and the Clinical Investigation Committee approved the study.

The enrollment period was based on the local respiratory virus epidemic wave progression. The cohort of patients were enrolled from 7 March 2020 to 10 February 2023. All patients were negative for SARS-CoV-2 infection. Patients who provided consent with diagnoses of lower respiratory infection, pneumonia, or respiratory failure were enrolled in the study within 48 h after hospital admission. Nasal samples were obtained from all participants by rubbing the nasal turbinates for 5 s with a cotton swab. Reverse transcription polymerase chain reaction (RT-PCR) on nasal samples was performed within 24 h of collection. *N* = 46 patients who were infected with RSV according to the PCR result are included in this analysis. Multiplex RT-PCR assay with Pathfinder (https://www.pathofinder.com/) was used to determine the co-infection status of the patients. This assay can simultaneously detect and differentiate between up to 18 different viral and 4 bacterial pathogens. The 18 viral agents were influenza A, Influenza B, RSV-A, RSV-B, Human metapneumovirus, and Rhinovirus/Enterovirus, Adenovirus, Influenza A H1N1n, Parainfluenza-1, Parainfluenza-2, Parainfluenza-3, Parainfluenza- 4, Bocavirus Type1, Coronavirus NL63, Coronavirus HKU1, Coronavirus 229E, Coronavirus OC43. The 4 bacterial agents were Mycoplasma pneumoniae, Chlamydia pneumoniae, Legionella pneumophila, and Bordetella pertussis.

Evaluations consisted of medical history, chart review, symptoms, physical findings laboratory, chest radiograph, or CT Scan. Steroid use was recorded for hospital admission or during hospitalization. Data collected from study questionnaires were summarized descriptively by pathogen and risk group. Patients were considered to be risk factors for progression to severe disease if they were aged ≥65 years or had COPD, Bronchial Asthma, Bronchiectasis Hypertension Obesity, Diabetes, Cardiac artery Disease (without leading to heart failure), Heart Failure, or Chronic Kidney Disease medical conditions. Acute respiratory symptom length before hospitalization and during hospitalization days were recorded. The patients were categorized according to their parenchymal infiltrations, oxygen saturation, and oxygen therapy during hospitalization.

### Statistical analyses and tests

All data analyses are carried out by R (https://www.rproject.org/). R functions used include *cor.test* for correlation (and test), *lm* for linear regression, *glm* for logistic regression (generalized linear regression with family = “binomial”). A basic introduction to linear and logistic regression is included in the Appendix of this article.

The power calculation was carried out by two programs. The first was the R package *pwrss* (https://cran.r-project.org/web/packages/pwrss/). The *pwrss.z.logreg* function was used. The second is the stand-alone R^*^Power program (version 3.1), downloaded from https://www.psychologie.hhu.de/arbeitsgruppen/allgemeine-psychologie-und-arbeitspsychologie/gpower. The “z test” was selected for the test family, and “logistic regression” for the statistical test.

## Results

### Summary statistics

The basic statistics of our 46 patients can be found in Table 1. There were more female patients (63%) than male (37%). Besides one patient who was 20 years old, four patients were in their 30s and the rest were middle-aged or seniors with a median age of 54.6 and a median of 52.5. The most common comorbidity includes hypertension (41%), asthma (33%), diabetes (30%), heart failure (20%), chronic kidney disease (11%), and cardiac disease (without leading to heart failure) (9%). There was only one case of bronchiectasis, and one case of obesity, and none of the patients had chronic obstructive pulmonary disease (COPD).

**Table 1:** Summary of demographic variable, comorbidities, symptoms, co-infection status, and time from onset of symptom to hospital visit (outpatients), time from admission to being without symptom (inpatients).

**Variable**	**Description**
Gender	F: 29 (63%), M: 17 (37%)
Age	52.5 (median) 54.6 (mean) 75 (max)
Smoking	1 (current) 9 (ever)
Hypertension	19/46 (41%)
Bronchial asthma	15/46 (33%)
Diabetes	14/46 (30%)
Heart failure chronic	9/46 (20%)
Kidney disease	5/46 (11%)
Cardiac disease	4/46 (9%)
Cough	41/46 (89%) 13/46 cough only
Fever	12/46 (26%)
Myalgia	9/46 (20%)
Dyspnea	8/46 (17%)
Weakness	8/46 (17%)
Co-infection	29/46 (63%), 7/46 (15%, influenza), 12/46 (26% bacterial)
Symptom time up to hospital visit	mean 8.3 (outpatients) 14.2 days (inpatients)
Inpatients symptom time	mean 22.8 days (mild-a), 20.3 (mild-b), 21.7 (moderate), 30.4 (severe)
Lung parenchymal infiltration	28 normal (61%) (mild-a)
Given oxygen	32 never given (26 normal plus 6 mild-b)
	7 received 2lt oxygen (moderate), 5 received 4lt (severe)

CT scan result and oxygen treatment status that are used to define the four disease severity groups.

In terms of symptoms, the most common ones included cough (89%), fever (26%), myalgia (20%), dyspnea (17%), and weakness (17%). The less common symptoms included sputum production (4 samples), and hemoptysis (2 samples).

Interestingly, the majority of the patients had co-infection (29 out of *n* = 46, or 63%). Among the 29 patients with co-infection, 12 were infected by bacteria whereas seven were infected by influenza. None of the patients who participated were infected with three – RSV, influenza, and bacteria.

Concerning disease severity, we classified the samples into four scales: mild-a, mild-b, moderate, and severe by the following definitions [for other classification schemes, see (41)].

- Mild-a: no parenchymal infiltration.
- Mild-b: parenchymal infiltrates present but no oxygen supplementation.
- Moderate: parenchymal infiltrates present, oxygen supplementation, and oxygen saturation 90 ~ 95.
- Severe: parenchymal infiltrates are present, oxygen supplementation, and oxygen saturation < 90.

There were 28 patients without lung parenchymal infiltration (mild-a class), though two of them received oxygen treatment due to a relatively low oxygen level (90 and 92) and had heart failure. There were six patients with infiltration who did not receive oxygen treatment (with oxygen levels of 90, 92, 93, 97, 98, and 98, respectively) (mild-b class). For the remaining samples, seven of them had oxygen levels in the 90s range (severe) and five of them had oxygen levels below 90. These two groups of patients received 2lt and 4lt oxygen treatment, respectively. The mean oxygen levels of these four severity classes were 96, 94.7, 91.3, and 85.6.

Our definition of disease severity was correlated but not identical to the hospitalization status of patients. Of our 19 inpatients, four were mild-a and three were mild-b. Of the 27 outpatients, three were mild-b, though none of them were moderate. The subdivision among inpatients in SARS-CoV-2 infected patients by disease severity has also been observed in another study (42).

The study observed that patients admitted to the hospital waited longer after the onset of symptoms [mean = 14.2 days, median = 14, range = (6,28)] than those who were not admitted [mean = 8.1, median = 6, range = (0,27)]. It is unclear if the delay in seeking professional help results in a more severe disease. Among patients admitted to hospital, we also recorded the length of their hospital stay (43). Taking into account the time patients waited before they went to hospital waiting time and hospital stay time, we defined a time from the onset of symptoms to hospital release (O2R). Based on this, there was little variation in time between the first symptoms and severity classes: for mild-a (*n* = 4), mild-b (*n* = 3), and moderate (*n* = 7) patients, the mean O2R times were 22.8, 20.3, and 21.7 days. However, for the severe patients (*n* = 5), O2R time was on average 30.4 days.

In terms of treatment, none of the *n* = 46 patients were admitted to ICU. Two patients required non-invasive mechanical ventilation. A total of 28 patients received steroid treatment. Among them, 10 received oral steroids, 12 received intravenous (IV) steroids, and 6 patients received both oral and IV steroids. The majority of the patients (40 out of 46, or 87%) received the Beta-2 adrenergic agonists medication by inhaler, and 43 out of 46 patients received the inhaler steroid for local effect on the bronchi. All inpatients were given short-term oxygen therapy. Finally, 13 patients were given antiviral drugs, and 21 patients were given antibiotic drugs.

### Summary statistics of the second order (correlation)

Second order summary statistics are another way to express “correlation”. Table 2 lists all pairs of variables that were statistically significant (at level 0.01) correlations in our dataset. With our relatively small sample size, to avoid false positives, we have marked *p*-values lower than 0.001 (instead of 0.01) in bold font. Even though the threshold setting of 0.001 is still arbitrary, it was argued on reasonable grounds that it is a good choice (44–47).

**Table 2:** Significant correlations among and between demographic variables, comorbidities, and symptoms.

**Var1**	**Var2**	***p*-value**	**Direction**
Gender	Smoking	**6.8E-5**	Male-smoking
Age	Smoking	**1.8E-4**	Older-smoking
Age	Gender	**1.5E-5**	Older-male
Hypertension	Heart	**3E-4**	+
Diabetes	Cardiac	0.009	+
Heart	CKD	0.0026	+
Gender	Hypertension	**5.8E-5**	Male-hypertension
Gender	Heart	**6.8E-5**	Male-heart
Gender	CKD	0.0092	Male-CKD
Smoking	Diabetes	0.0023	+
Smoking	Cardiac	**3.4E-4**	+
Age	Hypertension	**1.1E-7**	Older-hypertension
Age	Diabetes	**8E-4**	Older-diabetes
Age	Heart	**1.7E-4**	Older-heart
Age	CKD	0.0076	Older-CKD
Age	Cardiac	**2.5E-7**	Older-cardiac
Cough	Fever	**5.7E-4**	-
Cough	Myalgia	**2.6E-6**	-
Fever	Myalgia	0.0076	+
Age	Weakness	**1E-5**	older-weakness
Hypertension	Weakness	**9.2E-4**	+
Diabetes	Weakness	**5.9E-4**	+
Cardiac	Weakness	**1E-4**	+
Asthma	Cough	0.0037	-
Gender	Bacteria-infection	0.004	Male-bacteria
Age	Co-infection	**1E-5**	Older-coinfection
Age	Bacteria	**6.5E-5**	Older-bacterial-infection
Diabetes	Co-infection	0.0019	+
Heart	Bacteria	**4.4E-4**	+
Cough	Bacterial	**5.7E-4**	-
Fever	co-infection	0.0062	+
Fever	Bacteria	**4E-5**	+
Myalgia	Bacteria	**1.3E-5**	+

Being significant means either p-value smaller than 0.01 or 0.001 (in bold). The p-value is obtained from a χ^2^ test if two variables are binary, or from a t-test is one variable is binary and another variable is continuous.

**Figure 1:**
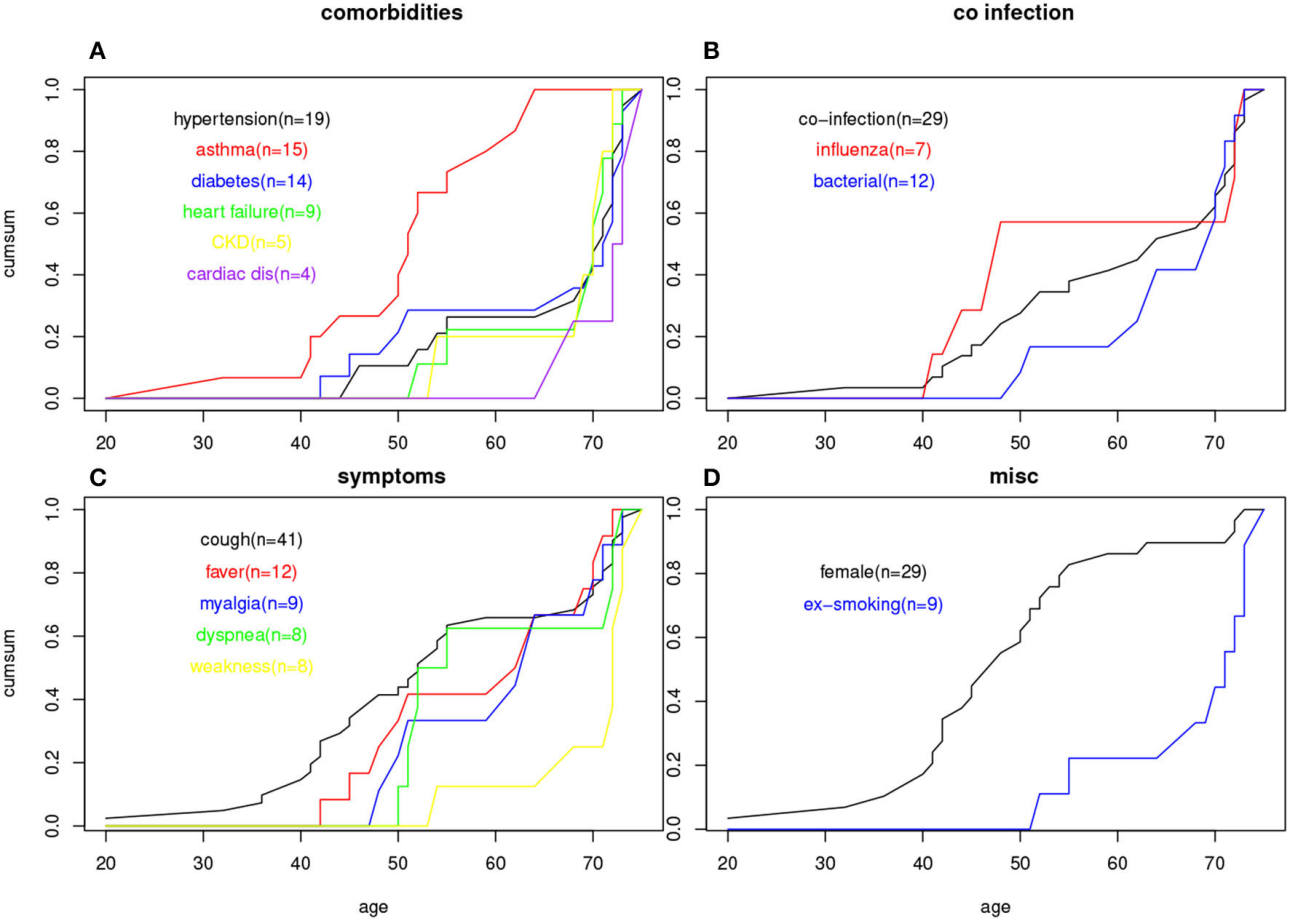
Age of onset cumulative plots for **(A)** six comorbidities; **(B)** three co-infection status; **(C)** five symptoms; **(D)** female gender and smoking status.

Table 2 shows that patients in our dataset tended to be older (mean age for male patients = 67.7 years and female patients = 48.4 years); active smokers tend to be male and older. Both of these trends can also be seen from the age of onset plot in Figure 1D. There is a positive correlation between hypertension and heart failure, between diabetes and cardiac diseases, and between chronic kidney disease and heart failure. As expected and well known, many pre-conditions/comorbidities are associated with male gender and older age, as listed in Table 2.

Concerning symptoms, fever and myalgia are positively correlated and both are negatively correlated with fever. Weakness is not only associated with older age, but also with several other pre-conditions including hypertension, diabetes, and cardiac disease. Coughing is the most prevalent symptom in our data (*n* = 41 out of 46), but only five patients without coughing have asthma. This leads to a negative correlation between asthma and coughing (but not significant at 0.001 level). We suspect that some asthma patients are so used to coughing that they may not answer the questionnaire correctly.

While older age patients tended to be more co-infected (as well as co-infected by bacteria), which may not be surprising, other patterns indicate that this hypothesis is worth further investigation. Patients with a pre-condition of diabetes are more susceptible to co-infection, and those with heart failure were associated with bacterial infection. To an extent, the co-infection status was associated with fever. Bacterial infection was also positively associated with fever and myalgia but negatively associated with coughing.

### Factors and symptoms associated with the disease severity

We carried out two regressions to determine which variables in Table 1 are associated with the disease severity. The first used linear regression, assuming a numerical level of 0, 1, 2, or 3 for mild-a, mild-b, moderate, and severe patients. Another is logistic regression using a binary severity level, 0 for mildred-a and mild-b, and 1 for moderate and severe. Although it is possible to have a binary severity level with the severe cases in one group and the rest in another, due to the small sample size in the severe group (*n* = 5), we did not obtain any statistically significant results at the *p* = 0.01 level.

Table 3 shows all factors or symptoms that are associated with the disease severity if *p*-value was < 0.01 (those with *p* < 0.001 are marked with bold font). The results from the two regressions are mostly consistent except for the co-infection factor, which had a significant result in linear regression but was non-significant for the logistic regression. All these associations were positive (increasing age and the presence of a factor were associated with a more severe disease).

**Table 3:** *P*-values from single-variable linear regression (dependent variable being the 4-level disease severity) and single-variable logistic regression (dependent variable being 1 for moderate and severe, 0 otherwise).

**Factor**	**pv(linearR)**	**pv(logisticR)**	**OR (95% CI)**
Age	**1.9E-4**	**0.001**	1.17 (1.06–1.28) per unit change of age
Hypertension	0.006	0.002	13.9 (2.5–75.9)
Diabetes	**2E-7**	**5.3E-4**	17.4 (3.4–87.5)
Heart failure	0.0038	0.0052	10.3 (2–53.2)
Myalgia	**2E-4**	0.034	
Co-infection	**8.7E-5**	0.99	
Bacterial infection	**1.4E-6**	**8.5E-4**	15 (3.1–73.6)

P-values lower than 0.001 are marked in bold. For logistic regression, the odds-ratio (OR) and its 95% confidence interval (CI) are shown. For continuous variables, e.g. age, OR refers to that between a unit change of the variable value.

Of the pre-conditions (comorbidities), hypertension, diabetes, and heart failure were associated with more severe disease, whereas asthma, chronic kidney disease cardiac disease were not. All *n* = 5 severe patients had muscle pain (myalgia), though none of the *n* = 7 moderate patients had the symptom. Although 39% of the mild-a cases had co-infection (*n* = 11 out of 17), 100% of the mild-b, moderate, and severe patients had co-infection. Similarly, among mild-a and mild-b cases, 12% had a bacterial infection, whereas, among moderate and severe cases, the proportion is 67%. The association between RSV infection severity and comorbidities (48) or co-infection (49) has been widely investigated and our results can be considered as another confirmation.

### Joint contribution of diabetes and another condition

Expanding from the single variable regression, it is natural to ask the question: which two variables contribute to disease severity independently? To examine this, we carried out a two-variable regression for all combinations of two factors in Table 1. Due to the similar results between linear regression with 4 severity scales and logistic regression with 2 levels (Table 3), we only carried out the linear regression.

Table 4 shows all pairs of factors/symptoms that achieve a 0.01 significance level for both two variables in a linear regression for 4-scale disease severity (and those with *p* < 0.001, marked with bold font). Since symptoms cannot be causes for severity, if one of the variables in the variable-pair is a symptom, it lacks an interpretation power.

**Table 4:** *P*-values and regression coefficients of two-variable linear regression when both two variables are significant at 0.01 level (bold font if *p*-value is smaller than 0.001).

**Factor1**	**pv(fac1)**	**coef(fac1)**	**Factor2**	**pv(fac2)**	**coef(fac2)**
Age	8.8E-4	0.033	Myalgia	9.4E-4	1.126
Diabetes	**9.9E-7**	1.348	Myalgia	**8.7E-4**	0.981
Hypertension	**3.1E-4**	0.952	Myalgia	**1.1E-5**	1.497
Heart failure	0.0078	0.913	Myalgia	**4E-4**	1.246
Co-infection	0.0023	0.9	Myalgia	0.0056	0.989
Hypertension	**2.7E-4**	1.127	Cough	0.0022	−1.464
Heart failure	**6E-4**	1.292	Cough	0.0085	−1.225
Hypertension	0.0019	0.931	Fever	0.008	0.875
Diabetes	**6.5E-8**	1.55	Fever	0.006	0.714
Smoking	0.0072	1.053	Fever	0.0032	1.053
Diabetes	**1.6E-8**	2.025	Weakness	0.0098	−0.958
Bacterial infection	**2.2E-8**	1.801	Weakness	0.0019	1.01
Diabetes	**9.4E-9**	1.547	Heart failure	**1.2E-4**	1.07
Diabetes	**6.2E-9**	1.746	CKD	0.004	1.089
Diabetes	**6.3E-10**	2.019	Cardiac dis	**5.7E-4**	−1.55
Diabetes	**4.5E-10**	1.389	Bacterial infection	**2.8E-9**	1.353

Three interesting co-risk-factor pairs are of note, including diabetes and heart failure, diabetes and cardiac diseases, and diabetes and bacterial infection (see Table 4). However, the interplay between diabetes and other conditions is different in these situations. Patients with both cardiac disease and diabetes avoided the severe situation (*n* = 0 of 4). Patients with both heart failure and diabetes all ended up in the severe cases (*n* = 3 out of 3), though two other patients with just one condition also had the severe disease. Finally, patients with both diabetes and bacterial infection (*n* = 5, of ages 50, 51, 70, 71, and 73) are all in the severe group, and those with only one condition (either diabetes or bacterial infection, not both) completely avoided the severe situation (*n* = 0 out of 16).

### Age structure for different comorbidities

Since age is very much associated with many other factors, we examined the age structure for people with comorbidities. Table 5 shows the number of patients with each comorbidity in each of the three age groups: younger than 49, 50–64, and older than 65. With the exception of asthma, the rest of the common comorbidities—hypertension, diabetes, heart failure, chronic kidney disease, and cardiac disease, tend to occur in the older age groups, a result that is of no surprise.

**Table 5:** The age distribution of patients with one, two, three comorbidities.

**Comorbidity**	** ≤ 49 years old**	**50–64**	**≥65**	**Total**
Hypertension	2	3	14	19
Asthma	4	11	0	15
Diabetes	2	2	10	14
Heart failure	0	2	7	9
CKD	0	1	4	5
Cardiac disease	0	0	4	4
Hypertension + diabetes	0	0	10	10
Hypertension + heart	0	2	7	9
Hypertension + CKD	0	1	4	5
Hypertension + cardiac	0	0	4	4
Athma+diabetes	0	2	0	2
Athma+heart	0	2	0	2
Diabetes+heart	0	0	3	3
Diabetes+cardiac	0	0	4	4
Cardiac+CKD	0	0	4	4
Hypertension+ diabetes+cardiac	0	0	4	4
Hypertension+ heart + CKD	0	0	4	4
Hypertension+ diabetes+heart	0	0	3	3
Hypertension+ asthma+heart	0	2	0	2

Table 5 also shows the age structure for patients with two pre-conditions. There were two comorbidity combinations, such as hypertension/diabetes, hypertension/cardiac disease, diabetes/heart failure, diabetes/cardiac disease, and cardiac disease/chronic kidney disease, that occur only in the 65-older group. Patients with these three comorbidities, hypertension/diabetes/cardiac disease, hypertension/heart failure/chronic kidney disease, and hypertension/diabetes/heart failure, are all in the 65 and older group; whereas the two hypertension/asthma/heart disease patients were in the 50–64 age group. There were no patients with 4 or more comorbidities.

To address the potential limitation that the age group is a too simplistic approach to characterize the age structure, we examined the age-of-onset for each patient's comorbidity in a cumulative plot, shown in Figure 1A. Besides confirming the previous observation that asthma is more a younger patient's comorbidity, and cardiac disease a senior's comorbidity, more subtle differences between comorbidities associated with older ages can be detected; for example, diabetes had a slightly younger age of onset than those who had heart failure or kidney disease.

### Age structure for co-infection, co-infection plus a comorbidity

The co-infection status can also potentially be confounded by age (as well as comorbidities). Table 6 shows the age distribution for patients co-infected with any other virus/bacterial, co-infected with influenza, and co-infected with bacteria. It is clear that while influenza coinfection does not have an age trend, bacterial co-infection is restricted to the middle and older age groups. Figure 1B shows the same trend based on individual patients' age information, as bacterial co-infection starts from around age 50, while flu co-infection occurs at age 40.

**Table 6:** The age distribution of patients with a co-infection status (co-infected by any viruses/bacteria, by influenza, by bacteria); age distribution of patients with both bacterial co-infection and another comorbidity.

**Comorbidity**	** ≤ 49 years old**	**50–64**	**≥65**	**Total**
Co-infection	7	8	14	29
Influenza	4	0	3	7
Bacterial	0	5	7	12
Bac + hypertension	0	0	7	7
Bac +asthma	0	5	0	5
Bac+ diabetes	0	2	3	5
Bac + heart	0	0	7	7
Bac + CDK	0	0	4	4

To understand the best statistical signal for disease severity from the joint impact of diabetes and bacterial infection, we examined the age structure for bacterial coinfection and comorbidities more closely. Table 6 shows the age distribution of patients who have both bacterial co-infection and a comorbidity. All seven patients with both bacterial infection and hypertension belong to the ≥65 age group, thus their joint action can be surrogated by the age group. The same conclusion can be reached for bacterial coinfection and heart failure, bacterial coinfection, and kidney disease. The five patients with bacterial coinfection and asthma all belong to the middle age group. However, the joint action from bacterial coinfection and diabetes are not represented by the age group, as they are distributed in two age groups.

## Discussion

RSV is an important pathogen not only for older seniors aged over 65, but also for middle aged patients in the 50**–**64 years range (40, 50–52). The adult RSV patients admitted to hospital included in our study had a median age of 53~54, which is slightly younger than those described in other studies, due to the inclusion of many patients in their 40 s. Among our cohort, none of the patients were aged over 76 years, providing a good opportunity to investigate factors whose contribution might be overshadowed by the age variable.

The adult hospitalized patients with RSV infection aged ≥ 50 years included in our study tended to have other comorbidities. Identifying high risk comorbidities is important for determining the cost-effectiveness and impact of treating the disease, as well as in providing an understanding of the disease etiology. Our stratified analyses found that RSV hospitalization risk was higher among adults with certain chronic medical conditions (CMCs) compared with those without the corresponding conditions. However, CMC prevalence is also higher in older adults. This creates a confounding factor (53) between CMCs and older age.

The most significant comorbidities/CMC risks for RSV hospitalization in our data were heart failure, hypertension (at 0.01 level), and diabetes (at 0.001 level) (see Table 3). Age itself was also a significant risk for hospitalization. In principle, one can add gender and age as co-variates in a multiple regression to remove the effect of gender and age. In practice, however, with our limited sample size, and with the requirement for a minimum number of samples per variable (54), output from such a multiple regression should be read with care. The three significant comorbidities in Table 3, hypertension, diabetes, and heart failure, would have *p*-values of 0.3 and 0.07, 0.04, respectively, when gender and age are added to the logistic regression; though the OR remains large. For the three comorbidity pairs in Table 4, two pairs remain significant: diabetes and heart failure (pv = 1.4E-7 and 1.6E-4), and diabetes and cardiac failure (pv = 7.5E-9, 5.7E-6).

As reported in a number of previous studies, heart failure or cardiopulmonary diseases represent are severe risk factors for RSV patients (25, 55–58). Diabetes and hypertension are also known risks (30, 59, 60). During the COVID-19 pandemic, it was suggested that the chronic condition of diabetes was not as strong a risk factor for severe conditions as acute hyperglycemia or new-onset diabetes (43, 61–65). However, our study did not generate the data required to address this question, on the impact of chronic vs. acute high glucose on the disease severity of RSV infected patients.

A potentially interesting result indicated by the data was that all of the severe patients (*n* = 5) included in the study had both diabetes and bacterial infection, and vice versa. This 100% match between the two might be a chance event of our relatively small dataset, but it leads to the significant two-variate regression (with diabetes and bacterial infection as two variables) for both variables (Table 4). If diabetes or co-infection is not an independent cause of RSV severity, then only one of the variables would be significant, not the other. One suggestion might be that diabetic patients, once infected by RSV and bacteria, have a three-way exacerbation. Table 2 shows that bacterial infection is associated with heart failure. Based on this data, another hypothesis is that it is an indication of a joint impact from diabetes and heart failure. Furthermore, the co-significance of diabetes and bacterial infection for RSV severity remains after adding gender and age to the multiple regression (pv = 1.1E-8, 9.7E-9).

Although the *n* = 46 RSV patients were a relatively small sample, we checked that it was high enough to detect strong signals. By using two different power calculation programs (R package pwrss and G^*^Power program), we estimate that *n* = 36 total samples (imbalanced such that 30% had comorbidity and 70% did not have) are required to have a power of 0.8 with a type I error of 0.05 (details not shown) to detect an odds-ratio of 17 signal (which is the observed OR value for diabetes as an independent variable, see Table 3). The exact sample size estimation in a power analysis depends on the parameter chosen, but within our choices of parameters only <100 samples were needed.

Our age-of-onset plots show that heart failure has a later age of onset than diabetes and hypertension (Figure 1A), and bacterial co-infection tends to appear later than influenza co-infection (Figure 1B). This also supports the idea that these two factors impact the severity of risk: one is related to an early age, and the other is related to a later age. The first group is represented by diabetes and the second group could be either heart failure or bacterial infection.

A comparison of the ages of the five patients for both diabetes and bacterial coinfection shows that they were not all seniors (Table 6) Two patients were middle aged (50 and 51 years old, both female) in the severe group, who had both diabetes and bacterial infection. This finding is crucial to our main conclusion. In the literature, disease severity is almost always attributed to an older age. Risk attributed to comorbidities is either completely associated with the older age, or by itself can't explain the observed data. Our result indicates that a comorbidity plus another condition (here, the bacterial coinfection) might cause the disease severity in the middle aged group. Our result is not only a confirmation of the extra burden caused by bacterial co-infection (66–68), but also a more specific proposal on joint action by a co-morbidity with that bacterial co-infection.

Among the RSV patient symptoms, cough remains the most common one by a big margin (*n* = 41 over the next common symptom, fever *n* = 12). Cough also appears in all age group from the relatively younger ones. Fever and myalgia symptoms have the next younger age of onset (Figure 1C). Relatively fewer patients have both cough and fever, and *n* = 7 patients have fever but no coughing. This led to a negative correlation between cough and fever. Also seen from Figure 1C, dyspnea starts to appear in mid-50 patients and is completely absent for patients in 40 s or younger. Weakness is only associated with elderly with age of onset beyond 70 s. The percentage of patients with specific symptoms can be compared with other studies, e.g., we have more patients with cough and fever than (69), comparable percentage of cough patients but less fever patients compared to (70, 71).

In conclusion, although advanced age is an important indicator in patients with RSV infection, it is thought that attention should be paid in patients with diabetes, heart failure, and hypertension. In particular, we observe that even for middle-aged RSV patients, having a pre- condition of diabetes and being co-infected by bacteria can be a severity-causing combination. This information should be useful for RSV patient management and treatment.

## Data availability statement

The raw data supporting the conclusions of this article will be made available by the authors, without undue reservation.

## Ethics statement

The studies involving humans were approved by the University of Tokat Gaziosmanpasa Faculty of Medicine Research Subjects Review Board and the Clinical Investigation Committee. The studies were conducted in accordance with the local legislation and institutional requirements. The participants provided their written informed consent to participate in this study.

## Author contributions

Conceptualization, methodology, writing—original draft, and writing—review and editing: HS, SC, AU, and WL. Data curation: HS. Formal analysis: SC, AU, and WL. Investigation and supervision: HS and WL. All of the authors declare that they have all participated in the design, execution and analysis of the paper, and have read and agreed to the published version of the manuscript.

## Appendix

### Linear and logistic regression with one or two independent variables

The standard linear and logistic regression with a single independent variable *x* is defined by these two equations (where the dependent variable *y* is either a continuous or a binary variable):

(1) $$\begin{array}{c} y=a+bx\\ prob\left(y=1\right)=\frac{1}{1+e^{-\left(a+bx\right)}} \end{array}$$

The fitting equation can be easily extended to two variables *x*_1_ and *x*_2_ (or more variables):

(2) $$\begin{array}{c} y=a+b_{1}x_{1}+b_{2}x_{2}\\ prob\left(y=1\right)=\frac{1}{1+e^{-\left(a+b_{1}x_{1}+b_{2}x_{2}\right)}} \end{array}$$

The *p*-value(s) in these regression is from testing the null hypothesis that the parameter is zero. If a *p*-value is very small (e.g., smaller than 0.01 or smaller than 0.001), the null hypothesis is unlikely to be true. This indicates that the independent variable contributes to the dependent variable (or *x* and *y* are statistically associated). When the *p*-value is smaller than 0.001, we say the statistical association is significant at 0.001 level.
